# Identification of immune-related genes in diagnosing atherosclerosis with rheumatoid arthritis through bioinformatics analysis and machine learning

**DOI:** 10.3389/fimmu.2023.1126647

**Published:** 2023-03-09

**Authors:** Fuze Liu, Yue Huang, Fuhui Liu, Hai Wang

**Affiliations:** ^1^ Department of Orthopaedic Surgery, Peking Union Medical College Hospital, Peking Union Medical College and Chinese Academy of Medical Sciences, Beijing, China; ^2^ School of Clinical Medical, Weifang Medical University, Weifang, China

**Keywords:** rheumatoid arthritis, atherosclerosis, immune infiltration, diagnosis, machine learning

## Abstract

**Background:**

Increasing evidence has proven that rheumatoid arthritis (RA) can aggravate atherosclerosis (AS), and we aimed to explore potential diagnostic genes for patients with AS and RA.

**Methods:**

We obtained the data from public databases, including Gene Expression Omnibus (GEO) and STRING, and obtained the differentially expressed genes (DEGs) and module genes with Limma and weighted gene co-expression network analysis (WGCNA). Kyoto Encyclopedia of Genes and Genomes (KEGG) and Gene Ontology (GO) enrichment analysis, the protein–protein interaction (PPI) network, and machine learning algorithms [least absolute shrinkage and selection operator (LASSO) regression and random forest] were performed to explore the immune-related hub genes. We used a nomogram and receiver operating characteristic (ROC) curve to assess the diagnostic efficacy, which has been validated with GSE55235 and GSE57691. Finally, immune infiltration was developed in AS.

**Results:**

The AS dataset included 5,322 DEGs, while there were 1,439 DEGs and 206 module genes in RA. The intersection of DEGs for AS and crucial genes for RA was 53, which were involved in immunity. After the PPI network and machine learning construction, six hub genes were used for the construction of a nomogram and for diagnostic efficacy assessment, which showed great diagnostic value (area under the curve from 0.723 to 1). Immune infiltration also revealed the disorder of immunocytes.

**Conclusion:**

Six immune-related hub genes (NFIL3, EED, GRK2, MAP3K11, RMI1, and TPST1) were recognized, and the nomogram was developed for AS with RA diagnosis.

## 1 Introduction

Rheumatoid arthritis (RA) is a systemic autoimmune disease characterized by chronic inflammation that commonly affects individuals aged 50–60 years (1). Patients with RA experience symmetrical joint pain and swelling, which may lead to joint deformity and progressive joint damage (2).

RA patients also have an increased risk of cardiovascular morbidity and mortality (3). Atherosclerosis (AS), the accumulation of a fibrofatty lesion in the artery wall with the infiltration of immunocytes such as macrophages, T cells, and mast cells, is a potential reason for coronary and carotid artery disease (4, 5). Recent evidence suggests that there are similar pathological processes and risk factors in both RA and AS, with chronic inflammation and immune dysfunction being the most significant (5–9).

While the underlying mechanism linking RA and AS is still unknown, it is clear that both conditions involve chronic inflammation and immune infiltration. For example, AS is an inflammatory process that can lead to plaque rupture, thrombosis, and vessel occlusion (10, 11). In patients with RA, immunological processes can occur many years before diagnosis, during the pre-RA phase (12). Furthermore, many pathological processes of the artery wall in AS are reflected in RA synovial inflammation, including the infiltration of macrophages and type 1 T helper cells, which have secondary effects on the artery *via* mediators produced in the synovium (7). Therefore, identifying immune infiltration and associated inflammatory molecules may have early diagnostic efficacy for RA patients with AS, which is significant in avoiding severe cardiovascular consequences.

In this study, we downloaded RA and AS datasets from the Gene Expression Omnibus (GEO) database and screened for differentially expressed genes (DEGs) using Limma. We identified significant module genes *via* weighted co-expression network analysis (WGCNA) and performed functional enrichment analysis. We constructed a protein–protein interaction (PPI) network for the intersection genes and identified candidate genes using machine learning algorithms, including the least absolute shrinkage and selection operator (LASSO) and random forest (RF), and immune cell infiltration analysis. We evaluated the key immune-associated diagnostic genes for AS with RA using nomogram and receiver operating characteristic (ROC) curve assessments. This study is useful in screening immune-related diagnostic biomarkers for AS in RA patients.

## 2 Materials and methods

### 2.1 Data collection and data processing

**Figure 1:**
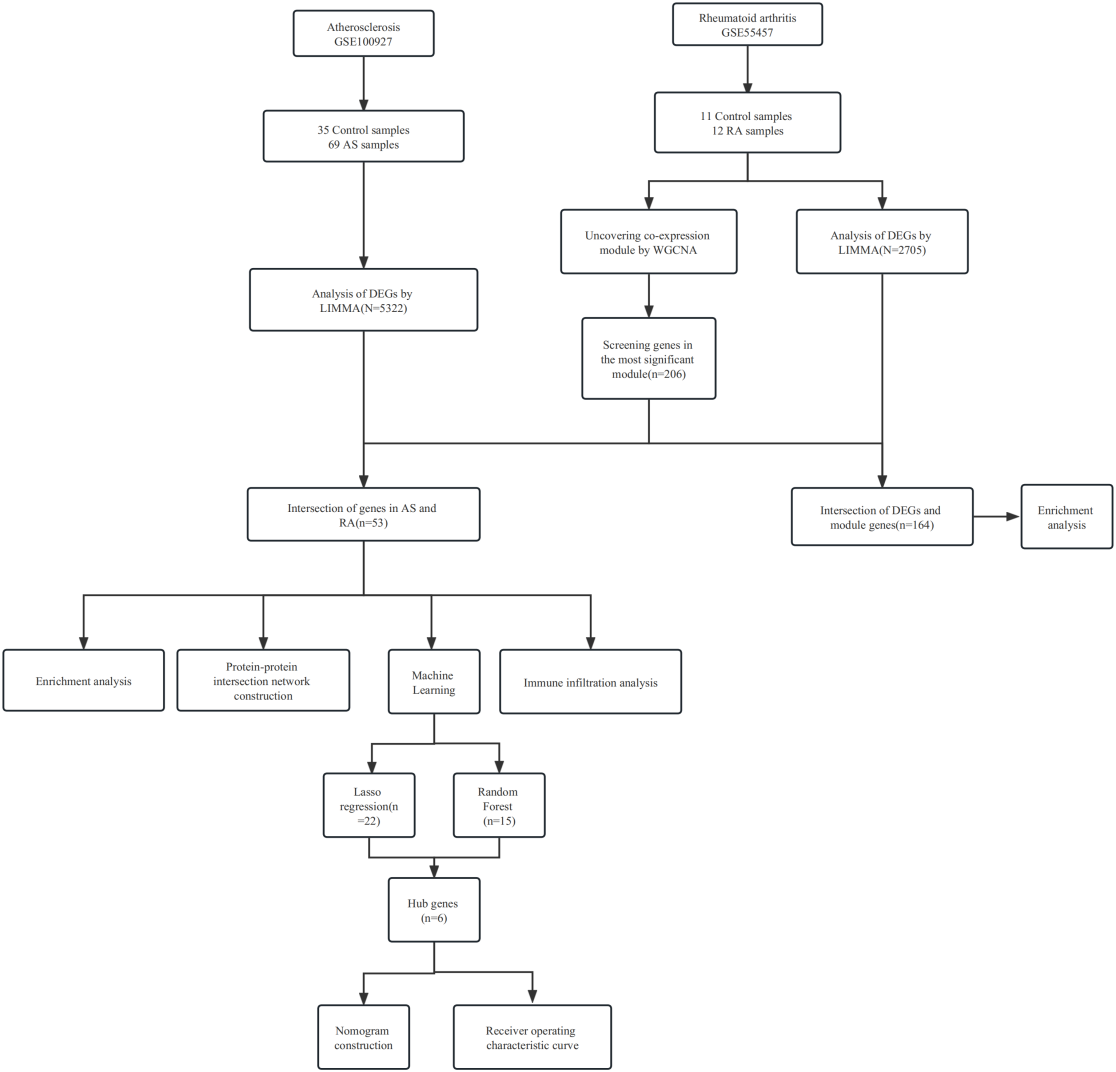
Workflow of the analysis.

We retrieved four gene expression datasets from the GEO database (https://www.ncbi.nlm.nih.gov/geo/), namely, GSE55457, GSE55235, GSE100927, and GSE57691 (13). The GSE55457 dataset included 11 control samples and 12 RA samples, while GSE55235 included 10 control samples and 10 RA samples. The GSE100927 dataset contained 35 control samples and 69 AS samples, and GSE57691 contained 10 control samples and 9 AS samples. We normalized the gene expression data using the R package “optparse.” The study procedures are summarized in **Figure 1**.

### 2.2 Differentially expressed gene screening

We obtained DEGs between RA and the control group with *p*_adj_ < 0.05 and |log_2_Fold change (FC)| > 1.2 in GSE55457, and between AS and the control group with *p*_adj_ < 0.05 and |log_2_FC| > 1.2 in GSE100927. The R software package Limma was used in this analysis. The DEGs were visualized *via* the Sangerbox platform (http://vip.sangerbox.com/).

### 2.3 Weighted gene correlation network analysis

In this study, we utilized the “WGCNA” package in R software to investigate the association between genes and phenotypes by constructing a gene co-expression network (14). Firstly, we removed 50% of genes with the smallest median absolute deviation (MAD). Secondly, we calculated Pearson’s correlation matrices for all pairwise genes and constructed a weighted adjacency matrix using the average linkage method and a weighted correlation coefficient. The “soft” thresholding power (*β*) was then used to calculate the adjacency, which was converted into a topological overlap matrix (TOM). To group genes with similar expression profiles into modules, we performed average linkage hierarchical clustering based on the TOM-based dissimilarity measure with a minimum gene group size of 50. Finally, we calculated the dissimilarity of module eigengenes, selected a cut line for the module dendrogram, and merged several modules. WGCNA was employed to identify significant modules in AS, and a visualized eigengene network was created.

### 2.4 Function enrichment analysis

To explore the biological functions of genes, we utilized the “clusterProfile” package in R software (15). First, we conducted Gene Ontology (GO) and Kyoto Encyclopedia of Genes and Genomes (KEGG) analyses, using a *p*-value < 0.05 (16, 17). The results were visualized using the Sangerbox platform (http://vip.sangerbox.com/). We then identified the intersection of DEGs in both AS and the critical module genes of RA, as well as the intersection of DEGs in RA and the critical module genes of RA. We performed GO and KEGG analyses based on these intersections.

### 2.5 Protein–protein intersection network construction

To investigate the interaction among proteins, pathways, and co-expression, we utilized the STRING database (https://cn.string-db.org/) to construct the protein–protein intersection (PPI) network of the DEGs for AS and the critical module genes (18). Cytoscape software was used to identify the significant interacted genes (19). Only the genes that interacted with each other were chosen for further analysis.

### 2.6 Machine learning

To further investigate the potential candidate genes for the diagnosis of AS with RA, we performed LASSO and RF analyses. LASSO, a machine learning technique that combines variable selection and regularization, can enhance predictive accuracy (20). On the other hand, RF is a predictive algorithm that does not impose restrictions on variable conditions, making it capable of providing predictions without apparent variations (21). We employed the R software’s “glmnet” and “randomforest” packages to conduct LASSO and RF analyses, respectively. The intersection of the two results can serve as the candidate hub genes for diagnosis (22, 23).

### 2.7 Nomogram construction and receiver operating characteristic evaluation

In order to determine the importance of the candidate genes for the diagnosis of AS with RA, we constructed a nomogram using the “rms” R package. The nomogram consisted of “Points,” which indicated the score of the candidate genes, and “Total Points,” which showed the total sum of all gene scores. The nomogram was an important tool for predicting the diagnosis of AS with RA. We further evaluated the prognostic value of the candidate genes and the nomogram by performing ROC analysis. The ROC analysis generated the area under the curve (AUC) and 95% confidence interval (CI), and an AUC value > 0.7 was considered to have great diagnostic efficacy.

### 2.8 Immune infiltration analysis

To estimate the infiltration of immune cells based on gene expression profiles, we utilized CIBERSORT, an analytical tool. We evaluated the proportion of immune cells in AS and control groups using this platform (24). The bar plot was used to visualize the proportion of various immune cells, while the vioplot was used to compare the proportions of these cells between the AS and control groups. The heatmap with Sangerbox platform was used to depict the association of immunocytes (25).

### 2.9 Statistical analysis

Statistical analysis was conducted to analyze the data obtained in this study. The ROC curve and AUC were constructed using SPSS Version 26.0 (IBM Corporation, Armonk, NY, USA), and the 95% CI was calculated. The proportion of various immunocytes between the RA and control groups was compared using the Mann–Whitney *U*-test *via* GraphPad Prism Version 8.3.0 (GraphPad Software, San Diego, CA, USA). A *p*-value<;0.05 was considered statistically significant.

## 3 Results

### 3.1 Identification of differentially expressed genes

**Figure 2:**
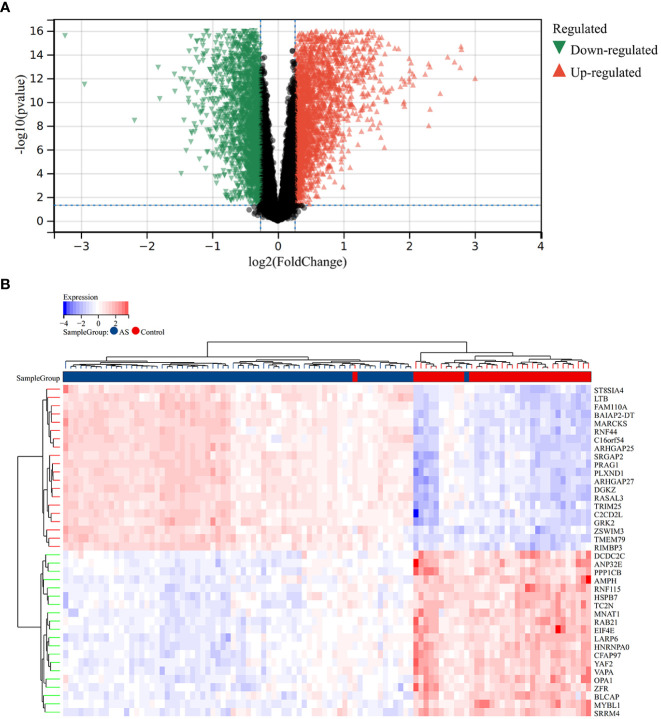
Different expression genes between AS and control groups. **(A)** Red and green represent DEGs with significantly higher and lower expression level in AS groups, respectively. **(B)** The heatmap showed the top 20 genes that were significantly expressed in the RA and control groups.

**Figure 3:**
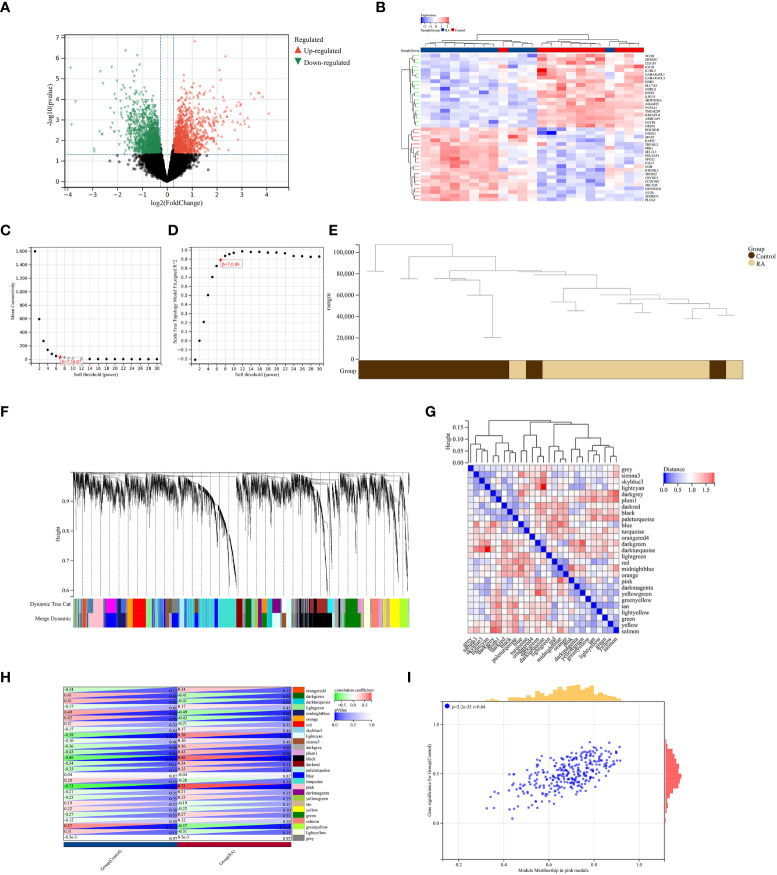
Identification of DEGs *via* Limma and WGCNA module genes in RA. **(A)** The volcano plot represents DEGs, of which the red and green triangles refer to significant genes. **(B)** The heatmap shows the top 20 upregulated and downregulated DEGs from the RA dataset, which are shown in red and blue colors. **(C, D)** *β* = 7 is chosen as the soft threshold based on the scale independence and average connectivity. **(E)** Clustering dendrogram of the RA and control samples. **(F)** Gene co-expression modules with different colors under the gene tree. **(G)** Heatmap of eigengene adjacency. **(H)** Heatmap of correlation between module genes and RA shows that the pink module has the highest association with RA. For each pair, the top left triangle is colored to represent the correlation coefficient; the bottom right one is colored to indicate the *p*-value. **(I)** Correlation plot between module membership and gene significance of magenta module genes.

A total of 2,705 DEGs were identified from the RA combined dataset with a *p*-value < 0.05 and |log2FC| > 1.2. The volcano plot and heatmap presented in **Figures 2A, B**, respectively, illustrate the differential expression pattern of these DEGs. Similarly, for AS, a total of 5,322 DEGs were identified using the same cutoff criteria of *p*-value < 0.05 and |log2FC| > 1.2. **Figures 3A, B** depict the differential expression pattern of these DEGs for AS.

### 3.2 Weighted gene co-expression network analysis and critical module identification

We constructed a scale-free co-expression network using the weighted gene co-expression network analysis (WGCNA) to identify the most associated module in RA. A “soft” threshold *β* of 7 was chosen based on the scale independence and average connectivity (**Figures 3C, D**). The clustering dendrogram of RA and control was generated, and 26 gene co-expression modules in different colors were obtained with a module merge threshold of 0.25 and a minimum size of 50, as shown in **Figures 3E–G**. Clinical correlation analysis results showed that the pink module had the highest association (*r* = 0.73, *p*-value < 0.001) with RA **Figure 3H**. Thus, we selected the pink module, which consisted of 206 genes, for further analysis. We conducted a correlation analysis between module membership and gene significance, and found a significant positive correlation between them (correlation coefficient = 0.64, *p*-value < 0.001) **Figure 3I**. These results indicated that the genes in the pink module were most closely related to RA.

### 3.3 Functional enrichment analysis of RA

**Figure 4:**
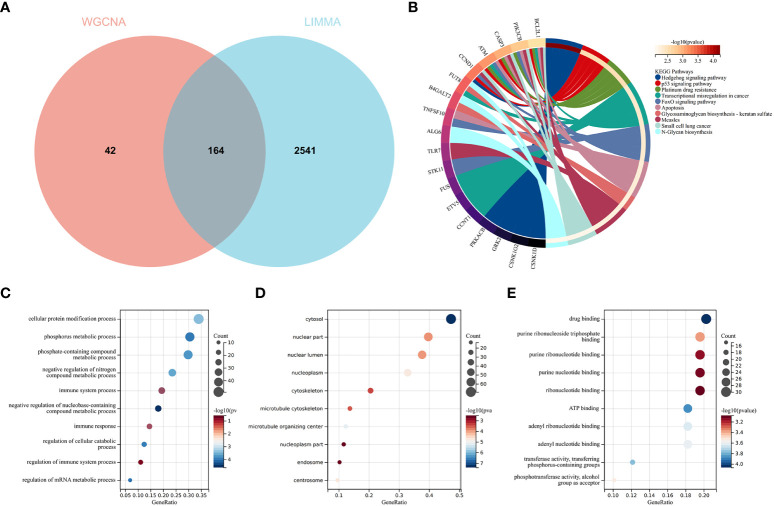
Function enrichment analysis of the intersection of genes for RA. **(A)** The intersection of DEGs *via* Limma and WGCNA module genes includes 164 genes, which were shown in the Venn diagram. **(B)** KEGG analysis of the intersection of genes. Various significant pathways and associated genes are represented with different colors. **(C–E)** The GO analysis includes biological process, cellular component, and molecular function. The *y*-axis represents GO terms, and the *x*-axis represents gene ratio involved in corresponding GO terms. The size of circles represents gene numbers, and their color refers to *p*-value.

To validate the reliable extent of GSE55457, we implemented enrichment analysis for the intersection of genes from Limma and module genes. A total of 164 common genes were obtained, as shown in **Figure 4A**.

KEGG analysis elucidated that common genes were involved in “p53 signaling pathway” and “Apoptosis”, as shown in **Figure 4B**. The results of GO analysis revealed that common genes were enriched in biological process (BP) terms, including “immune system process”, “immune response”, and “regulation of immune response”, as shown in **Figure 4C**. For cellular component (CC) ontology, the common genes are involved in “cytosol”, “nuclear part”, and “nuclear lumen”, as shown in **Figure 4D**. For molecular function (MF), the results showed that “drug binding” was the most significant term in common genes, as shown in **Figure 4E**.

The results showed that the common genes for RA were associated with immune response, which were highly related to the pathogenesis of RA.

### 3.4 Enrichment analysis of AS with RA and screening node genes *via* the protein–protein interaction network

**Figure 5:**
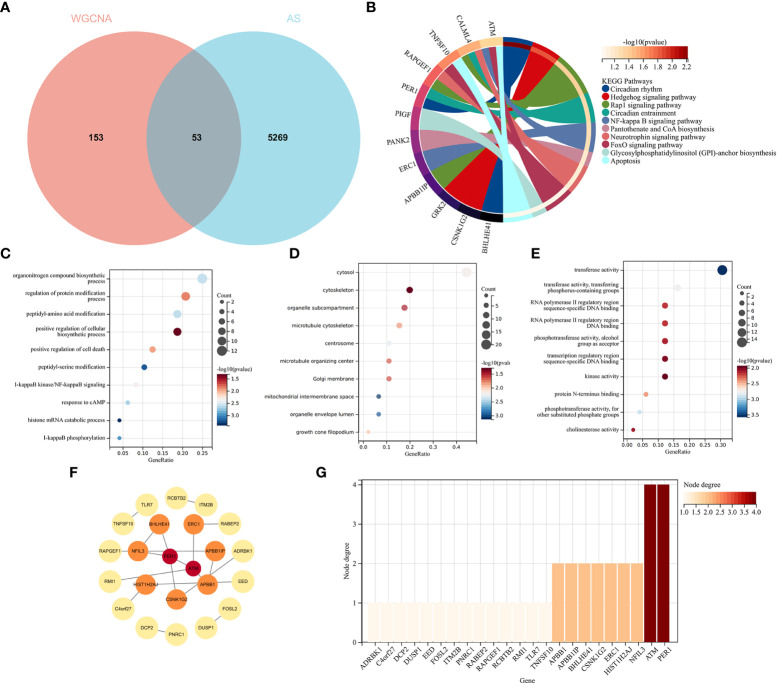
Functional enrichment analysis of common genes from RA with AS and the recognition of node genes with the PPI network. **(A)** Venn diagram shows 53 genes are recognized from the intersection of genes in RA with Limma and SLE with WGCNA. **(B)** KEGG analysis of 53 common genes. **(C–E)** GO analysis (biological process, cellular component, and molecular function) of 53 common genes. **(F)** The PPI network demonstrates that 23 genes interact with each other. **(G)** The column shows the gene nodes of 23 genes in the PPI network.

The intersection of the DEGs for AS and the module genes for RA included 53 genes, as seen in **Figure 5A**. To explore the relationship between RA-related genes with the pathogenesis of AS, enrichment analysis was performed based on these genes. The KEGG analysis showed that 53 genes mainly enriched in “NF-kappaB signaling pathway” and “Neurotrophin signaling pathway”, which were all closely associated with the immune system, as shown in **Figure 5B**. GO analysis revealed that genes were involved in “NF-kappaB signaling pathway”, “I-kappaB phosphorylation” (BP), “cytosol”, “cytoskeleton” (CC), and “transferase activity” (MF), as shown in **Figures 5C–E**.

A PPI network was constructed, in which 22 genes can interact with each other, as shown in **Figure 5F**. The node genes were ranked by node numbers in **Figure 5G**.

### 3.5 Identification of candidate hub genes *via* machine learning

**Figure 6:**
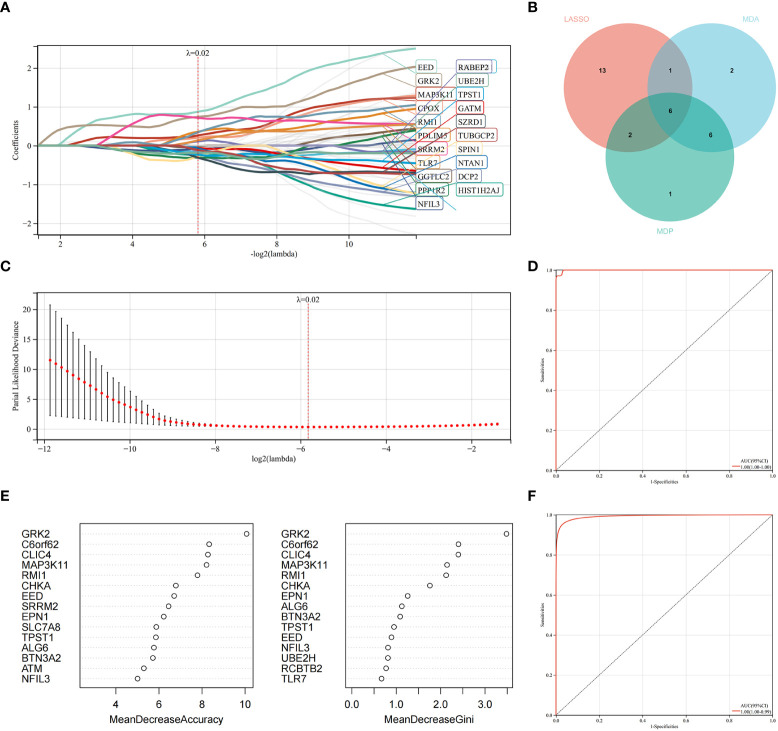
Machine learning in identifying key diagnosis genes for RA with AS. **(A, B)** Key genes identified in the LASSO model. Twenty-two genes are the most suitable for diagnosis. **(C)** The random forest algorithm ranks the top 15 most important genes based on MDA and MDP. **(D)** The intersection of genes of the above two algorithms is shown in the Venn diagram. **(E)** The ROC curve of the LASSO model. **(F)** The ROC curve of random forest algorithm.

LASSO regression and RF machine learning algorithms were utilized to identify potential candidate genes associated with the diagnosis of AS with RA. LASSO regression analysis identified 22 genes that were closely associated with the disease (**Figures 6A, B**). In the RF algorithm, we evaluated the importance of genes based on indicators such as mean decrease accuracy (MDA) and mean decrease gini (MDG) (**Figures 6C, D**). The AUC and 95% CI of these genes in LASSO regression and the intersection of MDA and MDG in RF machine learning algorithms were calculated, and the ROC curves were plotted. The results showed high accuracy for the LASSO regression (AUC 0.999, CI 0.971–1) and RF machine learning algorithms (AUC 0.995, CI 0.971–0.986) (**Figures 6E, F**). The intersection of the top 15 most important genes from RF and 22 genes from LASSO were visualized in **Figure 6E**, which identified six genes (NFIL3, EED, GRK2, MAP3K11, RMI1, and TPST1) as key diagnosis genes for the final validation.

### 3.6 Diagnosis value evaluation

**Figure 7:**
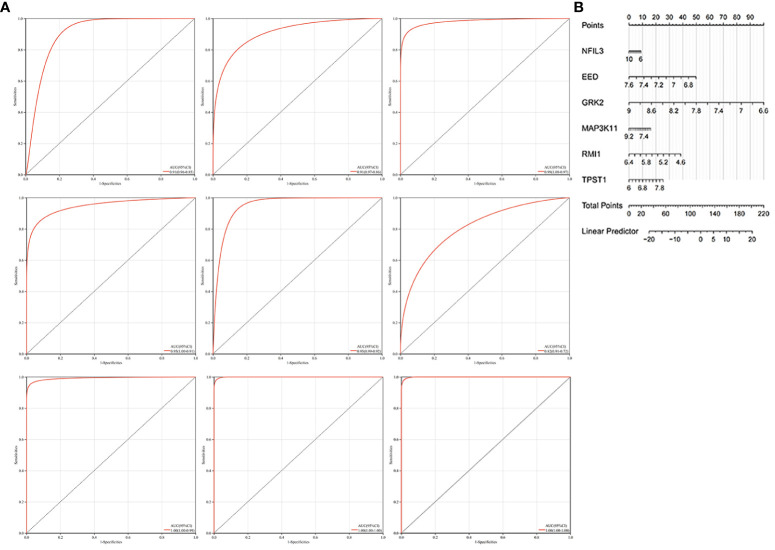
Construction of the nomogram and the diagnosis value assessment. **(A)** The ROC curve of each candidate gene (NFIL3, EED, GRK2, MAP3K11, RMI1, and TPST1), nomogram, and the validation in GSE55235 and GSE57691. **(B)** Nomogram for diagnosis RA with AS.

We constructed the nomogram with six key diagnosis genes, as shown in **Figure 7B**. The AUC and 95% CI of these genes were calculated with the construction of ROC curves to evaluate the diagnostic efficacy as shown in **Figure 7A**. The results were as follows: NFIL3 (AUC 0.907, CI 0.8515–0.9622), EED (AUC 0.915, CI 0.8582–0.9712), GRK2 (AUC 0.986, CI 0.9669–1), MAP3K11 (AUC 0.954, CI 0.9089–0.9984), RMI1 (AUC 0.953, CI 0.9157–0.9903), TPST1 (AUC 0.815, CI 0.723–0.9076), and nomogram (AUC 0.996, CI 0.9839–1). We validated the model with GSE55235 and GSE57691, as shown in **Figure 7A**. All genes and nomogram showed a high value of diagnosis for AS with RA.

### 3.7 Immune infiltration analysis

**Figure 8:**
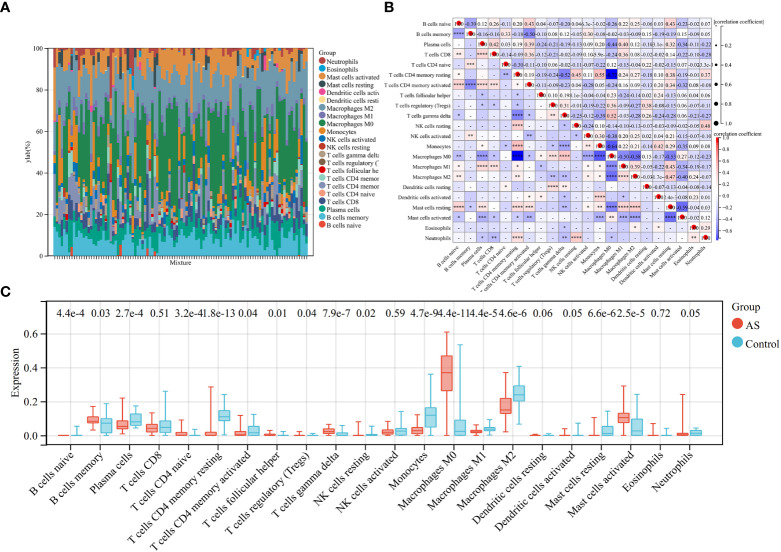
Immune infiltration analysis between AS and control. **(A)** The proportion of 22 immunocytes in all samples visualized from the bar plot. **(B)** Comparison of the proportion of 22 kinds of immunocytes between AS and control groups shown in the vioplot. **(C)** Association of 22 immunocyte-type compositions. *p < 0.05; **p < 0.01; ***p < 0.001.

Because the key diagnosis genes that were correlated with RA can regulate the pathogenesis of AS and be mainly enriched in immunity, the immune infiltration analysis can better explore the effect of immunity in AS. For AS and the control groups, the proportion of 22 kinds of immunocytes are shown in **Figure 8A**. The box plot presented that compared with the control group, naïve B cells, plasma cells, CD4+ naïve T cells, CD4+ memory-activated T cells, follicular helper T cells, activated NK cells, monocytes, M0 macrophage, M1 macrophage, M2 macrophage, resting mast cells, and activated dendritic cells had a lower level in the AS group, while memory B cells, regulatory T cells, gamma delta T cells, and activated mast cells had a high level, as shown in **Figure 8B**. The correlation of 22 types of immunocytes demonstrates that CD4+ memory resting T cells were positively related to monocytes (*r* = 0.55), monocytes were negatively related to M0 macrophage (*r* = −.64), CD4+ memory resting T cells were negatively related to M0 macrophage, (*r* = −0.72), and all the associations are shown in **Figure 8C**. In summary, the different level of infiltration of immunocytes in RA patients may serve as a potential treatment target.

## 4 Discussion

Accumulation of plaque in the artery wall, known as, is a primary cause of cardiovascular diseases and is closely associated with complications of the heart, brain, and kidney (26–28). Due to the difficulty in diagnosing and treating AS, finding an appropriate diagnostic biomarker is crucial to improve the prognosis (29). AS and RA share similar pathological processes, and the mortality rate of AS in RA patients is significantly increasing (30). Therefore, we performed bioinformatics analysis and machine learning methods to construct a nomogram to evaluate the diagnostic efficacy of AS in RA patients. We identified six key immune-related candidate genes (NFIL3, EED, GRK2, MAP3K11, RMI1, and TPST1) and constructed a nomogram.

Nuclear-factor interleukin 3 (NFIL3), also known as E4BP4, is a new biomarker for diagnosing AS in RA patients. NFIL3 exerts a transcriptional repressing function by binding to an activation transcription factor (ATF) DNA consensus sequence site (31). As a crucial transcription factor in the immune system, the expression level of NFIL3 is regulated by cytokines and mainly found in natural killer cells, B lymphocytes, T lymphocytes, and other immune cells (31–33). As a crucial transcription factor in the immune system, the expression level of NFIL3 is regulated by cytokines and mainly found in natural killer cells, B lymphocytes, T lymphocytes, and other immune cells (34–36). Inhibition of NFIL3 expression in CD4+ T cells decreases the level of IL10, worsening autoimmune encephalomyelitis (37). NFIL3 promotes the Th2 lineage while inhibiting the Th17 lineage and suppresses the production of IL-12 p40 in macrophages, which is associated with the progression of colitis (37, 38). Additionally, the anti-inflammatory effect of NFIL3 in immunity plays a crucial role in autoimmune diseases. NFIL3 has a high expression level in CD4+ T cells of patients with systemic lupus erythematosus (SLE) and suppresses the activation and self-reactivity of T cells and subsequent autoimmune response by downregulating CD40L (39). T follicular helper cells in patients with SLE also show a high level of NFIL3 but a low level of phosphorylation (40). Furthermore, the deficiency of NFIL3 is associated with juvenile idiopathic arthritis and induces more severe arthritis (41). The significant increase in NFIL3 in patients with RA may be associated with the production of multiple pro-inflammatory cytokines and RA progression (42). However, the association of NFIL3 with AS is still unclear. Due to the pro-inflammatory effect of NFIL3 in patients with RA, and the inflammation being a crucial factor in plaque rupture and stability, we suggest that NFIL3 could be a candidate diagnostic gene for AS in RA patients.

Embryonic ectoderm development (EED) is a nuclear factor and a transcriptional repressor. It is a member of the polycomb repressive complex and is involved in the proliferation and differentiation of lymphocytes as well as embryonic development (43–45). WAIT-1, a protein cloned from EED, interacts with integrins at the plasma membrane and plays a crucial role in immunity (46, 47). The activation of the integrin receptor can recruit EED to the plasma membrane, where it participates in the antigen receptor transduction in T cells (44, 48). EED also interacts with the neutral sphingomyelinase 2, which is involved in inflammation, heart failure, AS, and other biological processes (45, 49, 50). The production of ceramide *via* sphingomyelin hydrolysis is involved in the formation of atherogenic plaques, making the sphingomyelinase an important target in the treatment of AS (50). The production of ceramide *via* sphingomyelin hydrolysis is involved in the formation of atherogenic plaques, making the sphingomyelinase an important target in the treatment of AS.

G protein-coupled receptor (GPCR) kinase 2 (GRK2) is a key node in multiple signaling networks and interacts with various cellular proteins associated with signal transduction. This interaction further promotes signal propagation after GPCR activation (51). The signal transduction involves various cells’ activation, including endothelial cells. Excessive angiogenesis is an important factor in the development of inflammatory diseases, such as RA (52, 53). A high expression level of GRK2 has been detected in the synovial tissues of RA patients (51). It has been proven that GRK2 participates in the progression of AS. The mouse with a GRK2 deficiency demonstrates defective angiogenesis and increasing chemokine and adhesion molecules as AS progresses (54). Moreover, GRK2 is a potential upstream kinase for vinculin *via* mediating phosphorylation of vinculin, which further induces the disruption of the VE-cadherin/catenin complex, promoting the generation of atherogenesis (55). In this study, GRK2 is identified as one of the candidate diagnosis biomarkers for AS with RA.

Mitogen-activated protein kinase 11 (MAP3K11) is a potential target for immune treatment due to its expression in T cells and its regulatory role in T-cell activation and cytotoxicity (56). In addition, MAP3K11 is upregulated by mechanical stress and is associated with the differentiation of bone marrow stromal cells (57, 58). MAP3K11 has also been identified as a target for AS, as its inhibition can reduce the expression of key genes in coronary artery disease and the migration of vascular smooth muscle cells (59–61). It can also be used as a diagnosis marker.

RecQ-Medoayed Genome Instability 1 (RMI1) is crucial for maintaining genomic stability and regulates adipocyte hyperplasia to maintain energy stability (62). RMI1 is upregulated by obesity and high-glucose conditions and plays a role in maintaining genome integrity during replicative stress (63, 64).

Protein-tyrosine sulfotransferase 1 (TPST1) catalyzes the sulfuration of tyrosine residues within the acidic motif of polypeptides (65). It has been proven that TPST1 can regulate immune and inflammatory response through catalyzing sulfation (66) involved in regulating immune and inflammatory responses through tyrosine sulfation (67). Additionally, tyrosine sulfation contributes to monocyte recruitment, a major factor in AS development, making drugs inhibiting TPST1 favorable in AS treatment (68, 69). In this study, TPST1 is selected as a candidate diagnosis biomarker.

It has been identified that immune cells and inflammation play a crucial role in the pathogenesis of AS (70). The interactions between immunocytes and the production of pro-inflammatory and anti-inflammatory chemokines have an important influence in the plaque rupture (5, 71, 72). In AS patients and the animal models of AS, it has been observed that circulating monocytes are associated with the size and stage of plaque (73, 74). Monocytes can further differentiate into macrophages, the key component of plaque, and become foam cells after the accumulation (70). Dendritic cells also participate in the adaptive immune response to AS-associated antigens and the formation of foam cells, further promoting the development of AS (75, 76). Furthermore, Th1 cells are the main type of CD4+ T cells in AS, which produce a large number of pro-inflammatory cytokines, while Th2 cells can produce IL-13 and IL-5 to antagonize atherosclerosis (77–79). The expression level of Tregs has decreased with the progression of AS (80, 81). Moreover, B2 cells, which participate in antibody production, dependent on T cells, promote the progression of AS. In our study, naïve B cells, plasma cells, CD4+ naïve T cells, CD4+ memory-activated T cells, follicular helper T cells, activated NK cells, monocytes, M0 macrophage, M1 macrophage, M2 macrophage, resting mast cells, and activated dendritic cells had a lower level in AS patients, while memory B cells, regulatory T cells, gamma delta T cells, and activated mast cells had a high level in AS patients, consistent with previous studies. In summary, the study on immune manifestation and inflammatory cytokines can favor the diagnosis and treatment for AS.

## 5 Conclusion

In this study, we have successfully identified six immune-related hub genes (NFIL3, EED, GRK2, MAP3K11, RMI1, and TPST1) using bioinformatics analysis and machine learning algorithms. These genes have shown a potential to serve as diagnostic candidate genes for AS in RA patients. Furthermore, our study has also highlighted the immune dysfunction in AS with RA. We have also constructed a nomogram for diagnosing AS with RA, which can aid in clinical decision-making. Overall, our findings may provide new insights into the pathogenesis and diagnosis of AS with RA. Further validation studies are warranted to confirm the clinical relevance of these genes in AS with RA.

## Data availability statement

The datasets presented in this study can be found in online repositories. The names of the repository/repositories and accession number(s) can be found in the article/**Supplementary Material**.

## Author contributions

YH, FHL and HW participated in reviewing the articles. FZL wrote the manuscript. All authors contributed to the article and approved the submitted version.
