# Corrigendum: Identification of immune-related genes in diagnosing atherosclerosis with rheumatoid arthritis through bioinformatics analysis and machine learning

**DOI:** 10.3389/fimmu.2024.1400160

**Published:** 2024-04-16

**Authors:** Fuze Liu, Yue Huang, Fuhui Liu, Hai Wang

**Affiliations:** ^1^Department of Orthopaedic Surgery, Peking Union Medical College Hospital, Peking Union Medical College and Chinese Academy of Medical Sciences, Beijing, China; ^2^School of Clinical Medical, Weifang Medical University, Weifang, China

**Keywords:** rheumatoid arthritis, atherosclerosis, immune infiltration, diagnosis, machine learning

In the published article, the wrong dataset was used for the final validation of diagnostic efficacy. The correct dataset should be the atherosclerosis dataset GSE57691. This led to several errors in the article.

There was an error in **Figure 7** as published. The wrong dataset GSE73754 has been corrected to GSE57691 in the legend and the ninth ROC curve has been corrected in the Figure. The corrected **Figure 7** and its caption appears below.

**Figure 7:**
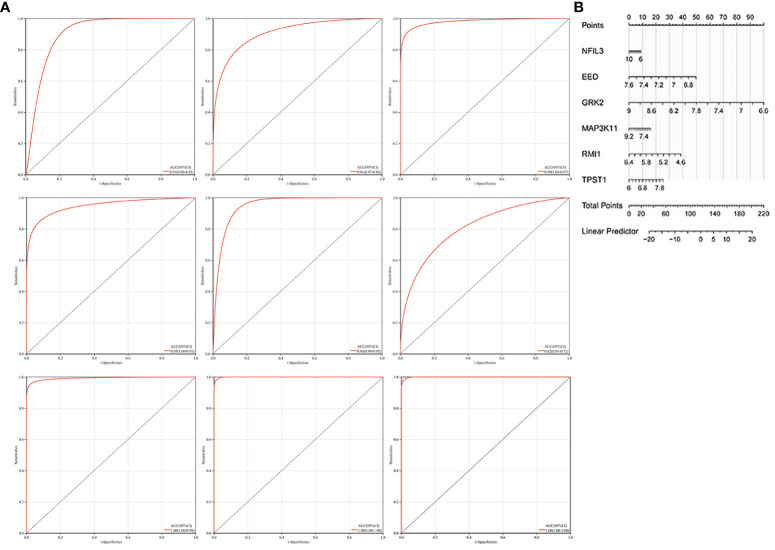
Construction of the nomogram and the diagnosis value assessment. **(A)** The ROC curve of each candidate gene (NFIL3, EED, GRK2, MAP3K11, RMI1, and TPST1), nomogram, and the validation in GSE55235 and GSE57691. **(B)** Nomogram for diagnosis RA with AS.

There was an error in **Supplementary Table 9**, Columns S and T. The information from the wrong data was mistakenly indicated in the supplementary material. The supplementary material has been corrected.

A correction has been made to **Abstract, Methods section,** Paragraph 8. This sentence previously stated:

“We used a nomogram and receiver operating characteristic (ROC) curve to assess the diagnostic efficacy, which has been validated with GSE55235 and GSE73754”.

The corrected sentence appears below:

“We used a nomogram and receiver operating characteristic (ROC) curve to assess the diagnostic efficacy, which has been validated with GSE55235 and GSE57691”.

A correction has been made to **2 Materials and methods, 2.1 Data collection and data procesing section,** Paragraph 3 and 7. This sentence previously stated:

“We retrieved four gene expression datasets from the GEO database (https://www.ncbi.nlm.nih.gov/geo/), namely, GSE55457, GSE55235, GSE100927, and GSE73754 (13). The GSE55457 dataset included 11 control samples and 12 RA samples, while GSE55235 included 10 control samples and 10 RA samples. The GSE100927 dataset contained 35 control samples and 69 AS samples, and GSE73754 contained 20 control samples and 52 AS samples”.

The corrected sentence appears below:

“We retrieved four gene expression datasets from the GEO database (https://www.ncbi.nlm.nih.gov/geo/), namely, GSE55457, GSE55235, GSE100927, and GSE57691 (13). The GSE55457 dataset included 11 control samples and 12 RA samples, while GSE55235 included 10 control samples and 10 RA samples. The GSE100927 dataset contained 35 control samples and 69 AS samples, and GSE57691 contained 10 control samples and 9 AS samples”.

A correction has been made to **3 Results, 3.6 Diagnosis value evaluation,** Paragraph 10. This sentence previously stated:

“We validated the model with GSE55235 and GSE73754, as shown in **Figure 7A**”.

The corrected sentence appears below:

“We validated the model with GSE55235 and GSE57691, as shown in **Figure 7A**”.

The authors apologize for this error and state that this does not change the scientific conclusions of the article in any way. The original article has been updated.

